# Antifungal Activity of Selenium Nanoparticles Obtained by Plant-Mediated Synthesis

**DOI:** 10.3390/antibiotics12010115

**Published:** 2023-01-08

**Authors:** Hugo Gerardo Lazcano-Ramírez, Jorge J. O. Garza-García, José A. Hernández-Díaz, Janet M. León-Morales, Alejandro S. Macías-Sandoval, Soledad García-Morales

**Affiliations:** 1 Department of Plant Biotechnology, Centro de Investigación y Asistencia en Tecnología y Diseño del Estado de Jalisco, Camino Arenero 1227, Zapopan 45019, Mexico; 2 Coordinación Académica Región Altiplano Oeste, Universidad Autónoma de San Luis Potosí. Carretera Salinas-Santo Domingo 200, Salinas de Hidalgo 78600, Mexico; 3 Department of Technological and Industrial Processes, Instituto Tecnológico y de Estudios Superiores de Occidente, Periférico Sur Manuel Gómez Morín 8585, San Pedro Tlaquepaque 45604, Mexico; 4 Department of Plant Biotechnology, CONACYT-Centro de Investigación y Asistencia en Tecnología y Diseño del Estado de Jalisco, Camino Arenero 1227, Zapopan 45019, Mexico

**Keywords:** phytopathogen, *Fusarium oxysporum*, *Colletotrichum gloeosporioides*, antimicrobial, SeNPs

## Abstract

The continuous need to satisfy world food demand has led to the search for new alternatives to combat economic losses in agriculture caused by phytopathogenic fungi. These organisms cause plant diseases, reducing their productivity and decreasing fruit quality. Among the new tools being explored is nanotechnology. Nanoparticles with antimicrobial properties could be an excellent alternative to address this problem. In this work, selenium nanoparticles (SeNPs) were obtained using plant extracts of *Amphipterygium glaucum* leaves (SeNPs-AGL) and *Calendula officinalis* flowers (SeNPs-COF). Characterization of the SeNPs was performed and their ability as antifungal agents against two commercially relevant plant pathogenic fungi, *Fusarium oxysporum* and *Colletotrichum gloeosporioides*, was evaluated. Assays were performed with different concentrations of SeNPs (0, 0.25, 0.5, 1.0, and 1.7 mg/mL). It was observed that both SeNPs had antifungal activity against both plant pathogens at concentrations of 0.25 mg/mL and above. SeNPs-AGL demonstrated better antifungal activity and smaller size (around 8.0 nm) than SeNPs-COF (134.0 nm). FTIR analysis evidenced the existence of different functional groups that constitute both types of SeNPs. There are factors that have to be considered in the antimicrobial activity of SeNPs such as nanoparticle size and phytochemical composition of the plant extracts used, as these may affect their bioavailability.

## 1. Introduction

Selenium (Se) is a semi-metallic element that belongs to the chalcogen family. Previously, it was considered toxic until it was found as an element with vital functions in some organisms such as mammals, archaea, algae, bacteria, and some insects and nematodes [1,2]. In nature, it exists in different oxidation states, inorganic forms such as selenite (Na_2_SeO_3_) and selenate (Na_2_SeO_4_), in the elemental state (Se^0^), and organic forms such as selenomethionine (SeMet) and selenocysteine (SeCyt) [3].

By itself, Se has important antimicrobial properties, as its effectiveness in inhibiting the growth of fungi and bacteria has been exposed [4,5]; for example, when used in sodium selenate (Na_2_SeO_4_), it was possible to completely inhibit the growth of *Fusarium* sp. and *Alternaria brassicicola* [4].

Efforts have been made to study the mechanisms of Se toxicity. However, this toxicity will depend in each case on the form of selenium applied and the response capacity of each organism. It has been observed that Se, in its organic and inorganic forms, has lower bioavailability and toxicity than in the form of nanoparticles (NPs) [2,6].

SeNPs have been synthesized mainly through chemical methods, using reagents such as sodium citrate [7] or sodium borohydride [8] and ascorbic acid [9]. The current investigations related to the biosynthesis of NPs are opening a new era of fast and non-toxic methods to produce NPs [10]

The search for new alternatives has led to exploring the use of natural plant products to synthesize NPs. Plants are rich in phytochemical compounds with important biological properties. About 25,000 terpenoids, 12,000 alkaloids, and 8000 known phenolic compounds have been identified [11]. The capacity and efficacy of phenols and terpenes as reducing agents of NPs in green synthesis have been demonstrated [12].

Different reports suggest that the composition of the plant extracts used for NP syntheses can change the geometry, morphology, or chemical composition of NPs [13], opening the door to a wide range of possibilities depending on the plant extract used.

The synthesis of nanoparticles using eco-friendly, low-cost methods that result in stable NPs is advantageous. The green synthesis of nanoparticles is a promising approach that utilizes natural extracts containing various bioactive molecules. Many parts of plants, including seeds, roots, flowers, and leaves, can be used as sources of compounds for the synthesis process [14].

There are reports on the synthesis with plant extracts of SeNPs mediated by tree gum as a stabilizer [8] as well as extracts from *Vitis vinifera* [15], *Ceropegia bulbosa* Roxb [16], and green orange peel [17]. The use of ascorbic acid as a reducing agent during the synthesis of SeNPs has also been reported [18]. 

In general, SeNPs are capable of inhibiting both Gram-negative and Gram-positive bacteria with equal efficacy. SeNPs have also been reported to exhibit antifungal activity, inhibiting spore germination [19]. The information on the capabilities and mechanisms of antimicrobial action of SeNPs is still limited today compared to other types of NPs, such as silver NPs (AgNPs), of which there is a broad overview of their mechanisms of action [2]. 

Currently, many investigations propose using NPs synthesized via green methods as an additional option to pest and disease management strategies in the agricultural sector [14]. There are various potential ways to incorporate SeNPs into agricultural practices, including adding Se to the soil, cultivating plants on a hydroponic or aeroponic nutrient medium containing SeNPs, soaking seeds in a solution of SeNPs before planting, or applying SeNP solution to plants through foliar application [20].

Worldwide, one of the main threats limiting agricultural crop production is diseases caused by phytopathogenic fungi. Among them, *Fusarium oxysporum* is one of the most common species of the genus *Fusarium*, is a ubiquitous soil-borne pathogen, and is characterized by causing vascular wilt of a wide number of economically important plant species [21]. *Colletotrichum gloeosporioides* is another pathogenic fungus associated with anthracnose symptoms in tropical fruit crops, affecting not only fruits but also leaves, flowers, and other plant organs. Anthracnose severely affects both production and marketing of agricultural products [22].

In this work, the antifungal and antibacterial activity of methanolic extracts of *Amphipterygium glaucum* leaves (AGL) and *Calendula officinalis* flowers (COF) was evaluated. With these extracts, via green synthesis, two types of NPs were previously obtained, SeNPs-AGL [23] and SeNPS-COF [24]; there is also a previous characterization of these SeNPs. In this study, a complementary characterization was performed and the ability of these NPs to inhibit the growth of two fungal species of economic importance, *Fusarium oxysporum* and *Colletotrichum gloeosporioides*, was assessed.

## 2. Results

### 2.1. Antimicrobial Activity of Plant Extracts

To identify the antimicrobial activity of extracts of *Amphipterygium glaucum* leaves (AGL) and *Calendula officinalis* flowers (COF), three phytopathogenic fungi and three Gram-negative bacterial strains with clinical involvement were used. The AGL extracts inhibited the growth of the fungus *Cladosporium cladosporioides* and the bacteria *Serratia marcescens*, *Enterobacter cloacae*, and *Alcaligenes faecalis*, resulting in a growth inhibition halo of about 9 mm (Table 1). COF extracts exhibited antibacterial activity against *S. marcescens* [24]. Benomyl (BEN), gentamicin (GEN), or ciprofloxacin (CIP) were used as positive controls; in all cases, there was a well-defined growth inhibition halo. The AGL and COF extracts exhibited no inhibitory effects on the growth of *F. oxysporum* or *C. gloeosporioides*. However, these extracts were used for the green synthesis of selenium nanoparticles because of their antimicrobial background and inhibitory effect on at least one microorganism evaluated.

### 2.2. SeNP Size

**Figure 1:**
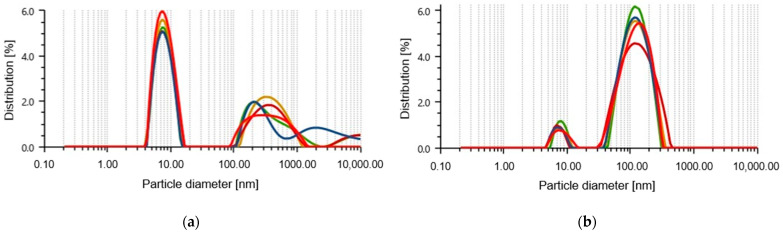
Size of selenium nanoparticles (SeNPs). (**a**) Nanoparticles synthesized with extracts of *Amphipterygium glaucum* leaves, called SeNPs-AGL. (**b**) Nanoparticles synthesized with extracts of *Calendula officinalis* flowers, called SeNPs-COF.

The size of the nanoparticles is a crucial characteristic of their antimicrobial activity. Using green synthesis, two types of SeNPs were obtained, one with *A. glaucum* leaf extracts (SeNPs-AGL) and the other with *C. officinalis* flower extracts (SeNPs-COF) (Table 2). With analysis of the particle size, it was found that the SeNPs-AGL present a peak with an average length of 8.08 nm (0.10 nm standard deviation, SD) and a peak 2 of 384.3 nm (66.66 nm SD), as well as 84.99 nm hydrodynamic diameter and 23.6% polydispersity index. The SeNPs-COF had a predominant mean size of 133.98 nm (6.31 nm SD), in addition to a 104.01 nm hydrodynamic diameter and 26.9% polydispersity index (Figure 1).

A previous characterization of the size using a transmission electron microscope (TEM) allowed us to identify sizes of 20–60 nm for SeNPs-AGL [23] and 40–60 nm for SeNPs-COF [24]. Via the TEM, it was obtained that both SeNPs had a close size range (≈20–60 nm); however, with the Litesizer, it was possible to identify that there were differences in size relative to an order of magnitude, which could influence the biological activity of SeNPs.

### 2.3. FTIR Analysis of SeNPs

**Figure 2:**
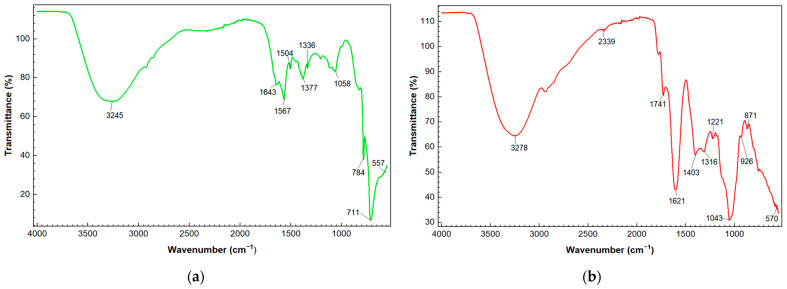
FTIR spectroscopy analysis. (**a**) FTIR spectrum of SeNPs-AGL synthesized with A. *glaucum* leaf extract. (**b**) FTIR spectrum of SeNPs-COF synthesized with *C. officinalis* flower extract.

From the FTIR analysis of the SeNPs, similar absorption bands were identified at 3245 cm^−1^ for SeNPs-AGL and 3278 cm^−1^ for SeNPs-COF, which would correspond to a stretching of the hydroxyl group (-OH) of the polyphenolic compounds existing in the plant extracts used for the synthesis of the SeNPs. Particularly, the SeNPs-COF absorption bands were obtained at 2339, 1741, and 926 cm^−1^. Meanwhile, the SeNPs-AGL had a specific absorption band at 1504 cm^−1^. In addition, other very close absorption bands can be observed in the two types of sintered SeNPs, such as 1557 vs. 1621, 1377 vs. 1403, 1058 vs. 1043, and 784 vs. 871 for SeNPs-AGL and SeNPs-COF, respectively (Figure 2).

### 2.4. Elemental Composition of SeNPs

**Figure 3:**
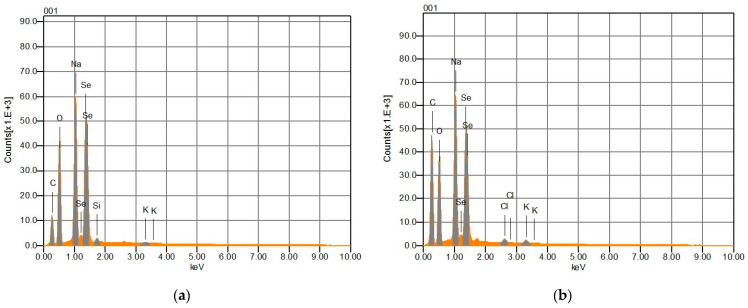
EDS analysis of the elemental composition of selenium nanoparticles (SeNPs) obtained via green synthesis. (**a**) SeNPs-AGL synthesized with extracts of *A. glaucum* leaves. (**b**) SeNPs-COF synthesized with *C. officinalis* flower extracts.

For the analysis of the elemental composition of the SeNPs, energy-dispersive X-ray spectroscopy (EDS) analysis was performed. Se, C, O, and Na were observed with high intensity in both the SeNPs-AGL and the SeNPS-COF (Figure 3). The presence of Na can be attributed to the source of Se (Na_2_SeO_3_) used to synthesize the SeNPs, while the C and O signals could correspond to the phytochemical compounds of the extracts of *A. glaucum* and *C. officinalis* used for the synthesis of SeNPs. EDS analysis revealed that SeNPs-AGL have a higher mass percentage of Se, O, and Na than COF-SeNPs. The latter SeNPs have almost twice the C mass percentage than SeNPs-AGL (Table 3). This could be explained by the final volume of the nanoparticle synthesis reaction and by the fact that the SeNPs-COF contain ascorbic acid as a reducing agent and the *C. officinalis* extracts as reaction stabilizers during the synthesis of the SeNPs [25].

EDS analysis revealed that SeNPs-AGL had a higher mass percentage of Se, O, and Na than COF-SeNPs. The latter SeNPs had almost twice the C mass percentage than SeNPs-AGL (Table 3). This could be explained by the final volume of the nanoparticle synthesis reaction and by the fact that the SeNPs-COF contain ascorbic acid as a reducing agent and the *C. officinalis* extracts as reaction stabilizers during the synthesis of the NPs [24].

### 2.5. Antifungal Activity of SeNPs

**Figure 4:**
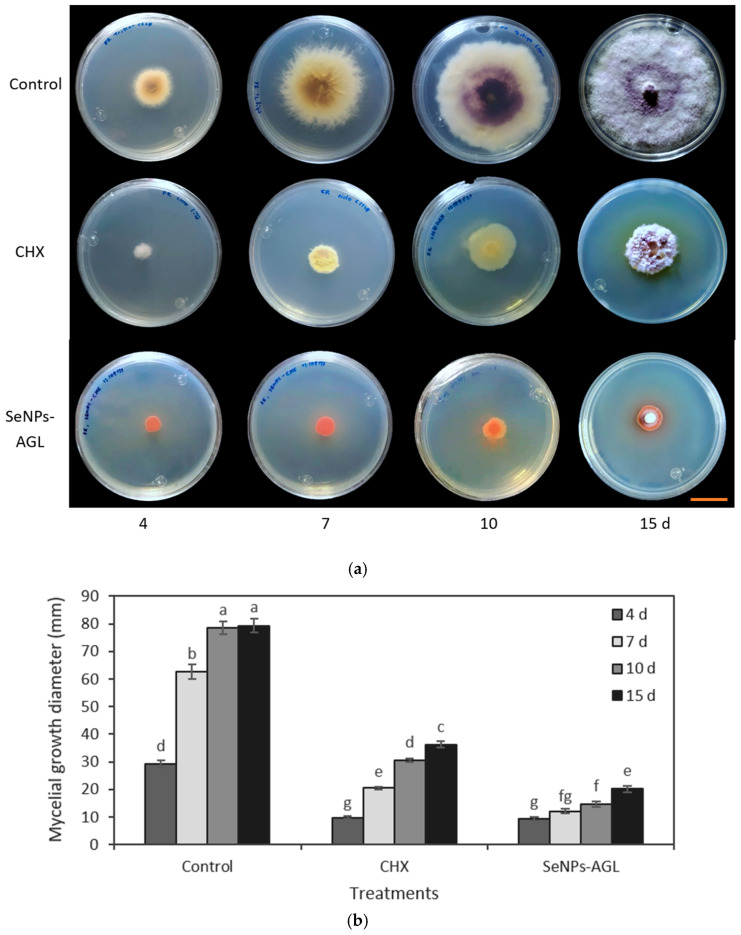
Antifungal activity of SeNPs synthesized with *A. glaucum* (AGL) extracts against *F. oxysporum*. (**a**) Representative photograph of the antifungal activity test under different treatments over time. SeNPs and CHX were applied at 1.7 mg/mL and 2.5 mg/mL, respectively. The scale bar is 2.5 cm. (**b**) Comparison of means of the effect of the SeNPs synthesized with extracts of *A. glaucum* against *F. oxysporum*. Mean values ± ee. Different letters indicate statistically significant differences according to Duncan’s test (α = 0.05), *p* < 0.0001.

**Figure 5:**
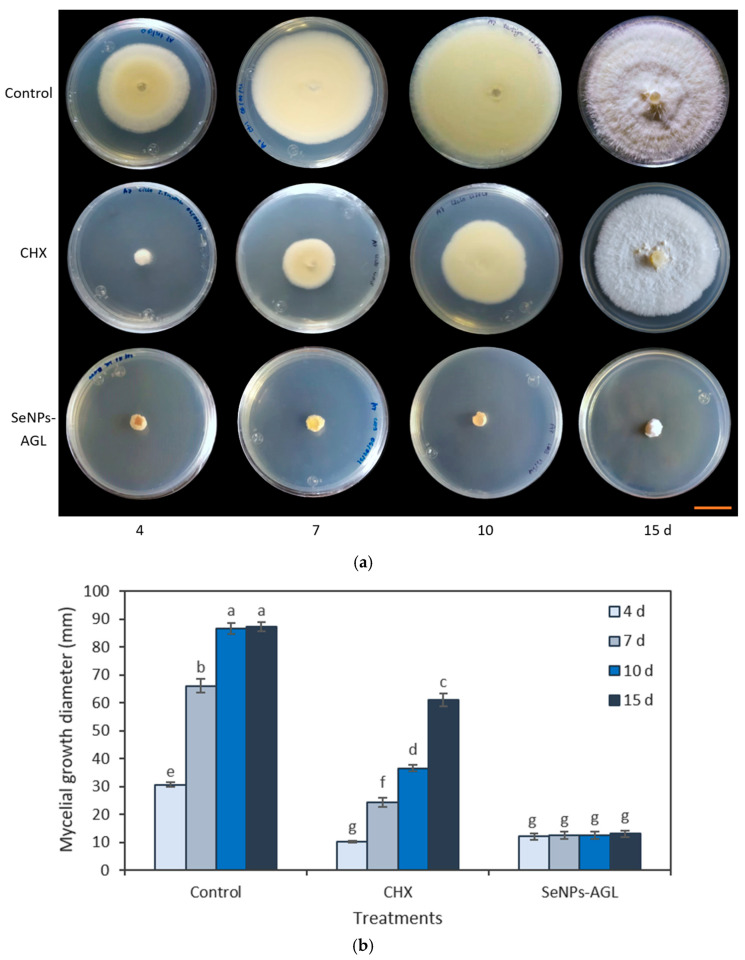
Antifungal activity of SeNPs synthesized with *A. glaucum* (AGL) extracts against *C. gloeosporioides*. (**a**) Representative photograph of the antifungal activity test under different treatments over time. SeNPs and CHX were applied at 1.7 mg/mL and 2.5 mg/mL, respectively. The scale bar is 2.5 cm. (**b**) Comparison of means of the effect of the SeNPs synthesized with extracts of *A. glaucum* against *C. gloeosporioides*. Mean values ± ee. Different letters indicate statistically significant differences according to Duncan’s test (α = 0.05), *p* < 0.0001.

**Figure 6:**
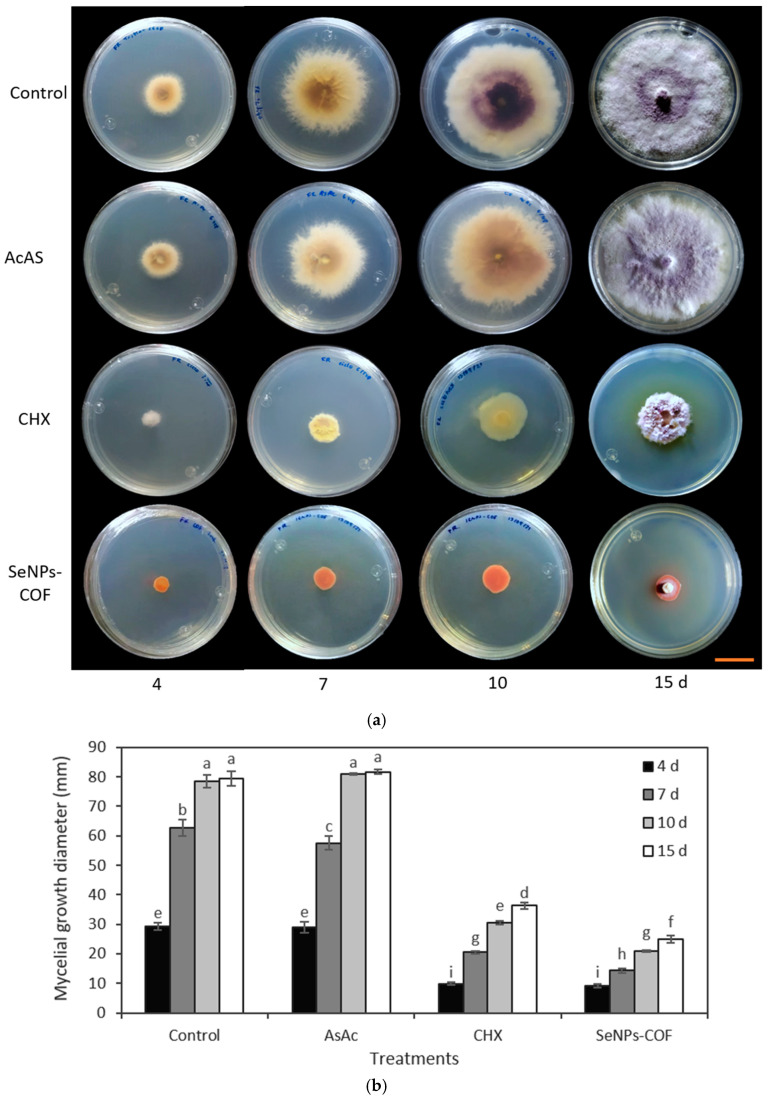
Antifungal activity of SeNPs synthesized with *C. officinalis* (COF) extracts against *F. oxysporum*. (**a**) Representative photograph of the antifungal activity test under different treatments over time. SeNPs and CHX were applied at 1.7 mg/mL and 2.5 mg/mL, respectively. The scale bar is 2.5 cm. (**b**) Mean comparison of the effect of the SeNPs synthesized with extracts of *C. officinalis* against *F. oxysporum*. Mean values ± ee. Different letters indicate statistically significant differences according to Duncan’s test (α = 0.05), *p* < 0.0001.

**Figure 7:**
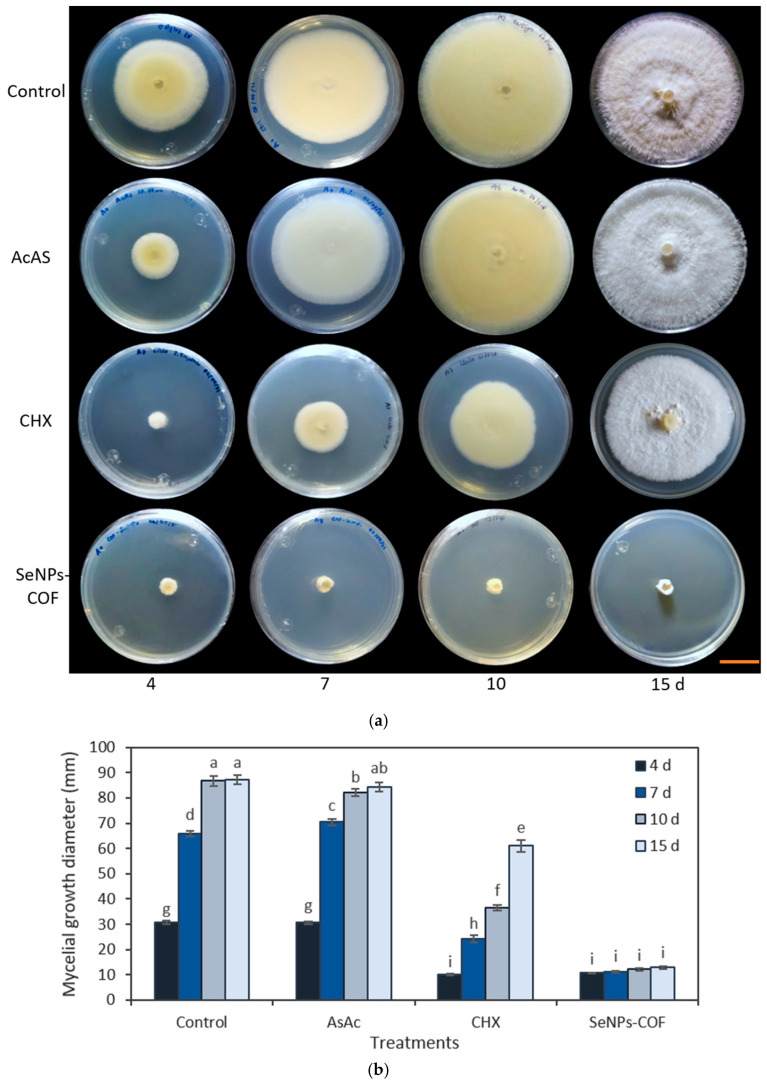
Antifungal activity of SeNPs synthesized with *C. officinalis* (COF) extracts against *C. gloeosporioides*. (**a**) Representative photograph of the antifungal activity test under different treatments over time. SeNPs and CHX were applied at 1.7 mg/mL and 2.5 mg/mL, respectively. The scale bar is 2.5 cm. (**b**) Mean comparison of the effect of the SeNPs synthesized with extracts of *C. officinalis* against *C. gloeosporioides*. Mean values ± ee. Different letters indicate statistically significant differences according to Duncan’s test (α = 0.05), *p* < 0.0001.

The medium poisoning technique was used to determine the antifungal activity of the SeNPs synthesized from methanolic extracts of *A. glaucum* against the fungi *F. oxysporum* (Figure 4) and *C. gloeosporioides* (Figure 5), as well as the antifungal activity of SeNPs synthesized from methanolic extracts of *C. officinalis* against these two phytopathogenic fungi (Figure 6 and Figure 7). First, the antifungal activity was evaluated using SeNP concentrations of 1.7 mg/mL and 1.28 mg/mL for *A. glaucum* (Figure 4 and Figure 5) and *C. officinalis* (Figure 6 and Figure 7), respectively, comparing them with the broad-spectrum antifungal cycloheximide (CHX). An untreated control was included in the assay, as well as ascorbic acid, to determine whether this component, used in the synthesis of SeNPs-COF, had any antifungal effect by itself.

The SeNPs of *A. glaucum* caused a statistically significant decrease in the growth of *F. oxysporum* from the seventh day in relation to the control and CHX (Figure 4). In the case of *C. gloeosporioides*, a similar trend was observed with a noticeable antifungal effect from the seventh day to fifteen days (Figure 5).

For the test of ascorbic acid (AsAc), it was confirmed that the effect of this component on *F. oxysporum* and *C. gloeosporioides* is practically null (Figure 6 and Figure 7). Antifungal activity of the SeNPs was observed from the fourth day until the end of the assay for both *F. oxysporum* (Figure 6) and *C. gloeosporioides* (Figure 7).

### 2.6. Effect of SeNP Concentration

**Figure 8:**
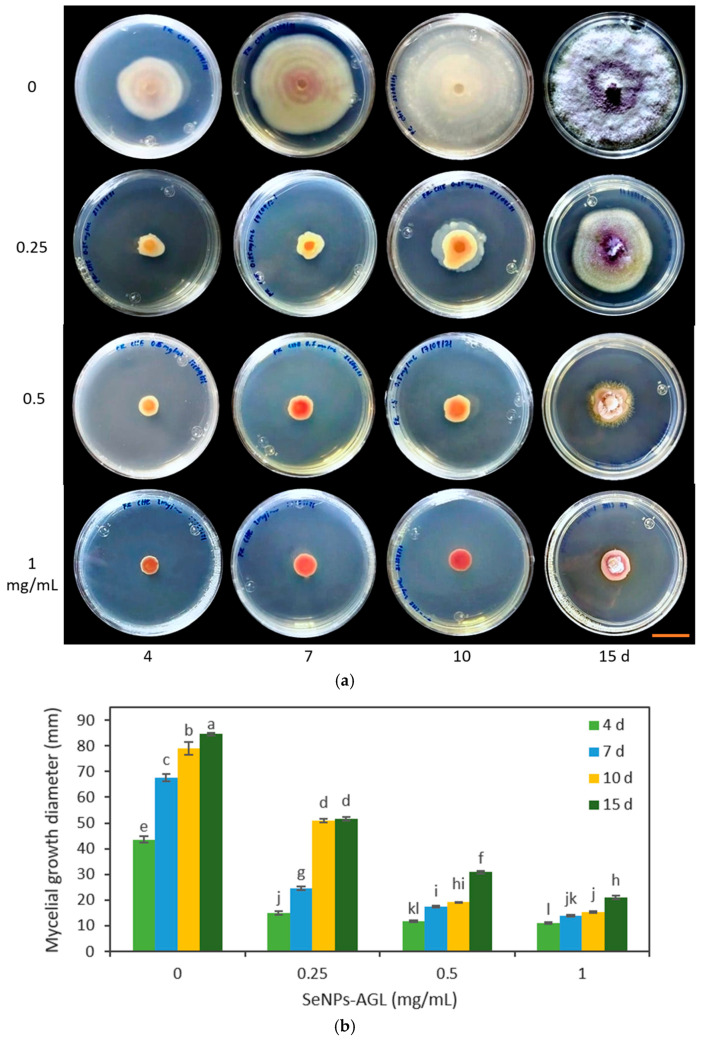
Effect of different concentrations of SeNPs synthesized with *A. glaucum* (AGL) extracts against *F. oxysporum*. (**a**) Representative photograph of the antifungal activity test under different concentrations over time. SeNPs were applied at 0.25, 0.5, and 1 mg/mL. The scale bar is 2.5 cm. (**b**) Mean comparison of the effect of the SeNPs synthesized with extracts of *A. glaucum* against *F. oxysporum* at different concentrations. Mean values ± ee. Different letters indicate statistically significant differences according to Duncan’s test (α = 0.05), *p* < 0.0001.

**Figure 9:**
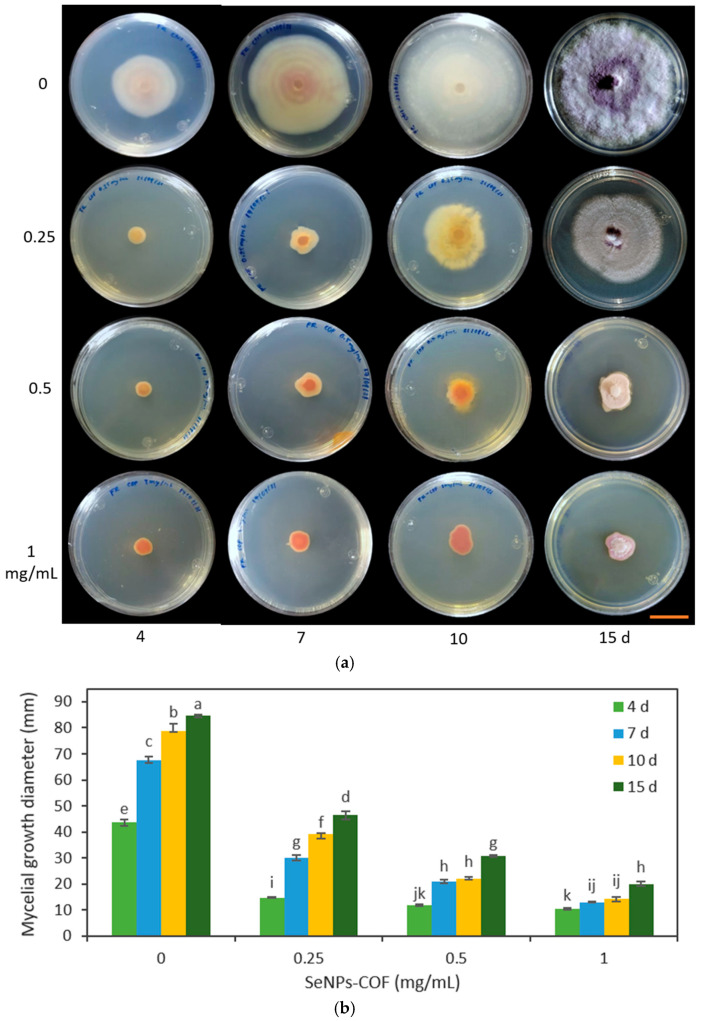
Effect of different concentrations of SeNPs synthesized with *C. officinalis* (COF) extracts against *F. oxysporum*. (**a**) Representative photograph of the antifungal activity test under different concentrations over time. SeNPs were applied at 0.25, 0.5, and 1 mg/mL. The scale bar is 2.5 cm. (**b**) Mean comparison of the effect of the SeNPs synthesized with extracts of *C. officinalis* against *F. oxysporum* at different concentrations. Mean values ± ee. Different letters indicate statistically significant differences according to Duncan’s test (α = 0.05), *p* < 0.0001.

In the second trial, the effect of different concentrations of SeNPs was verified through the evaluation of SeNPs at 0.25, 0.5, and 1.0 mg/mL on the growth of *F. oxysporum* and *C. gloeosporioides*. It should be noted that the changes in the *Fusarium* coloration were visible under the four concentrations used in both types of SeNPs (Figure 8 and Figure 9).

**Figure 10:**
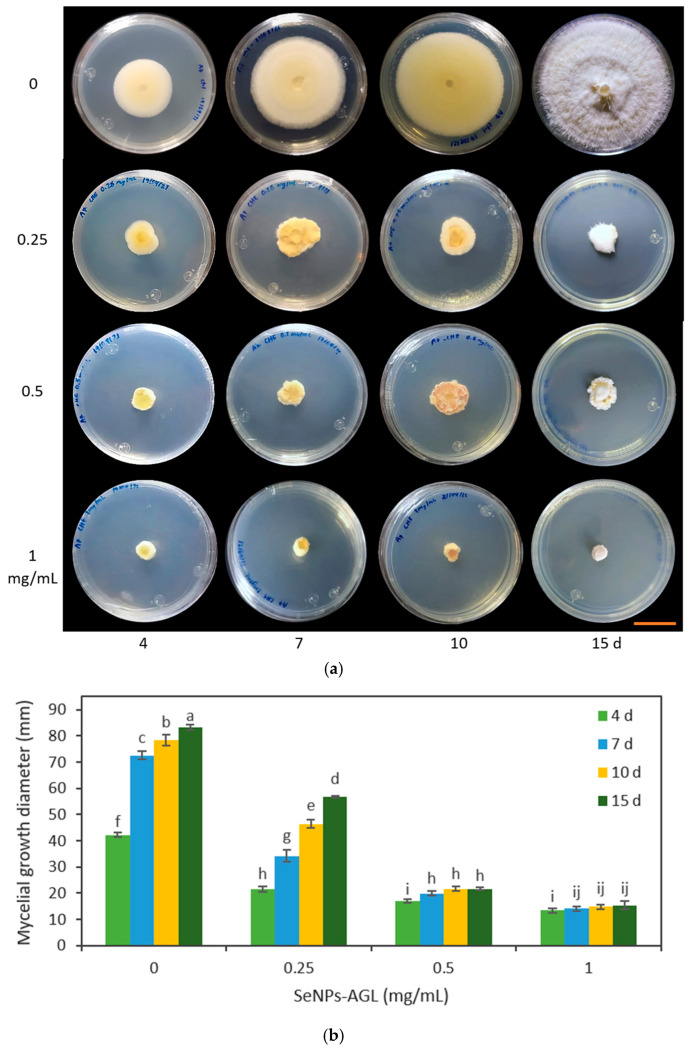
Effect of different concentrations of SeNPs synthesized with *A. glaucum* (AGL) extracts against *C. gloeosporioides*. (**a**) Representative photograph of the antifungal activity test under different concentrations over time. SeNPs were applied at 0.25, 0.5, and 1 mg/mL. The scale bar is 2.5 cm. (**b**) Mean comparison of the effect of the SeNPs synthesized with extracts of *A. glaucum* against *C. gloeosporioides* at different concentrations. Mean values ± ee. Different letters indicate statistically significant differences according to Duncan’s test (α = 0.05), *p* < 0.0001.

**Figure 11:**
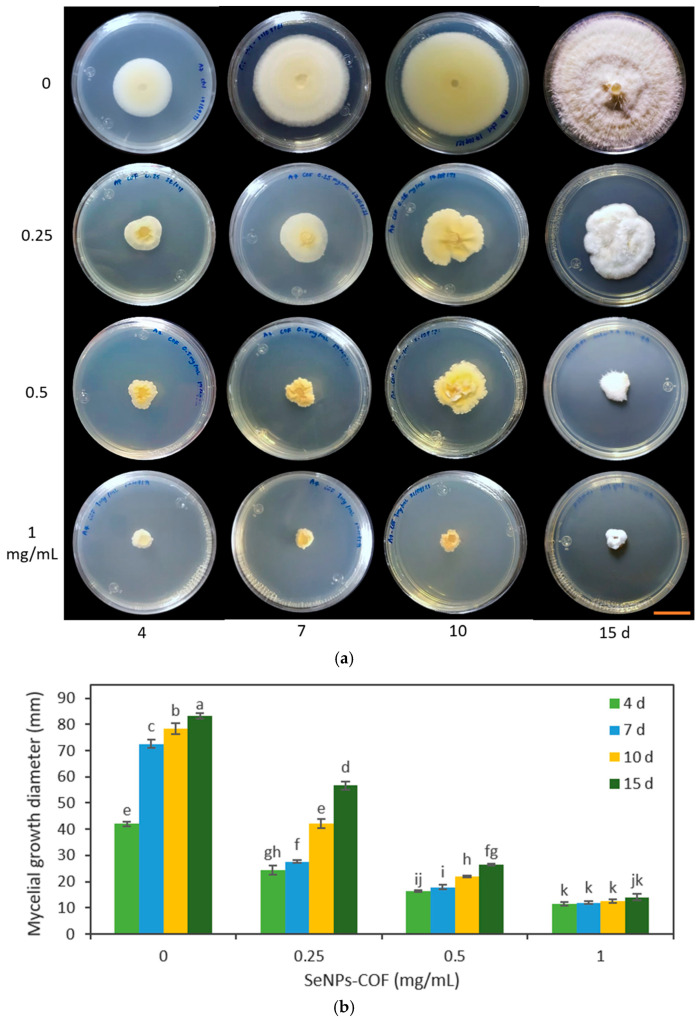
Effect of different concentrations of SeNPs synthesized with *C. officinalis* (COF) extracts against *C. gloeosporioides*. (**a**) Representative photograph of the antifungal activity test under different concentrations over time. SeNPs were applied at 0.25, 0.5, and 1 mg/mL. The scale bar is 2.5 cm. (**b**) Mean comparison of the effect of the SeNPs synthesized with extracts of *C. officinalis* against *C. gloeosporioides* at different concentrations. Mean values ± ee. Different letters indicate statistically significant differences according to Duncan’s test (α = 0.05), *p* < 0.0001.

The SeNPs derived from *C. officinalis* and *A. glaucum* were applied against *F. oxysporum*. It was found that the three concentrations tested (0.25, 0.5, and 1 mg/mL) demonstrated an antifungal effect from 4 d (Figure 8 and Figure 9). Of the different treatments, the concentration of 1 mg/mL maintained the same percentage of reduction in the growth of the fungus. This agrees with the data obtained with the concentration of 1.28 mg/mL; at these concentrations, the SeNPs were not able to completely inhibit the mycelial growth of the fungus, but stopped its growth compared to the other treatments. The antifungal effect shown by these SeNPs on the growth of *C. gloeosporioides* demonstrated a growth inhibitory effect for all the concentrations used, equally visible from 4 d (Figure 10 and Figure 11).

The application of the SeNP treatments (0.25, 0.5, and 1.0 mg/mL) produced morphological changes in the *C. gloeosporioides* colonies with both types of NPs. A change in the morphology of the base of the fungus was observed, going from being smooth to an amorphous one without well-defined edges. This effect was visible with the highest concentrations of SeNPs used (Figure 10 and Figure 11).

## 3. Discussion

Because there is currently a global trend towards the use of antimicrobial substances with reduced adverse effects on human health, plant extracts from plants that have been used throughout history due to their therapeutic properties are ideal candidates to begin exploring their potential use in producing these safer alternatives, especially for those substances that will be used in products for human consumption.

The *Amphipterygium* genus has curative properties such as healing, antibiotic, hypocholesterolemic, anti-inflammatory, antifungal, and antiprotozoal. It has also been used to dissolve gallstones and kidney stones and eliminate colic, fever, and digestive tract cancer. It is known as the most important anti-ulcer remedy in traditional Mexican medicine [26]. It also has potential use in areas such as agriculture and medicine, where its use has involved obtaining extracts to synthesize NPs. Such is the case of the study driven by Rodríguez-Luis et al. [27], who synthesized AgNPs from the metabolites of *A. adstringens* and verified the bactericidal and antifungal activity against the growth of *E. faecalis* and *C. albicans*.

There are several reports on the antimicrobial activity of *A. adstringens*; however, there is scarce information on *A. glaucum*. In this work, it was found that *A. glaucum* leaf extracts (AGLs) formed growth inhibition halos against the fungus *C. cladosporioides* and the bacteria *S. marcescens*, *E. cloacae*, and *A. faecalis* (Table 1). The major pharmacological properties of *A. adstringens* have been reported in extracts from the stem bark [26], whereas in this investigation, extracts were obtained from the leaves without affecting the stem of the plant.

*Calendula officinalis* has a long history of use in medicine and floriculture, making it one of the most important cultivated plants globally [28]. Calendula has a wide range of compounds such as carotenoids, phenolic acids, flavonoids, steroids, terpenoids, mucilages, saponins, coumarins, essential oils, and fatty acids, which may be present in different parts of the plant [29]. Due to its phytochemical composition, there are reports on the antimicrobial activity of *C. officinalis*. Previously, it was reported that *C. officinalis* flower extracts (COFs) had antibacterial activity against *S. marcescens* [24]. In this work, no antifungal activity was found.

Two types of SeNPs (AGL and COF) were obtained via green synthesis using extracts from *A. glaucum* leaves and *C. officinalis* flowers. It is expected that SeNPs with different characteristics will be obtained, since the phytochemical composition is different in each part of the plant and in each plant species. It is known that organic compounds from plants act as reducers and stabilizers of nanoparticles, and secondary metabolites play a crucial role in the formation of nanoparticles [30]. In the case of the SeNPs-AGL, the *A. glaucum* extracts fulfilled the reducing and stabilizing functions for forming SeNPs. Meanwhile, in the SeNPS-COF, in addition to the flower extracts of *C. officinalis*, ascorbic acid was used as a reducing agent (Table 2), as has been used for the green synthesis of other SeNPs [18]. These conditions resulted in nanoparticles with different characteristics in size, shape, and biological activity.

In a previous characterization, via TEM analysis, it was identified that the SeNPs-AGL have a spherical morphology with a size of 20 to 60 nm with antibacterial and biostimulant activity for plant growth [23]. To evaluate the antifungal activity, the size of the SeNPs was determined with a different instrument (Litesizer). It was found that the SeNPs-AGL present two peaks (Figure 1a), and the highest distribution percentage corresponds to an average size of 8.08 nm, significantly lower than that obtained by SEM (Table 2). The second peak corresponds to an average size of 384.3 nm (Figure 1a), which could correspond to the size of the nanobars previously observed by TEM for these nanoparticles [23]. Hernández-Díaz et al. [24] reported a size of SeNPs-COF of 40–60 nm and a spherical morphology via TEM, while, in this work, an average size of 133.98 nm was found. In a lower distribution percentage, a peak corresponding to a size of 7.74 nm was observed (Figure 1b).

The discrepancy between the size of SeNPS obtained via SEM versus via Litesizer is due to the fact that the latter is based on the dynamic light scattering (DLS) technique. The results obtained can be reasonably accurate for the case of strictly monodisperse NPs, since DLS does not allow distinguishing between NPs with slight differences in diameter, nor does it accurately resolve polydisperse samples. This is because the DLS method measures the intensity of the scattered light, which is proportional to the sixth power of the particle diameter. In a polydisperse sample, the scattered light from larger particles or agglomerates is strongly superimposed on that from smaller particles. Compared to other analytical methods, DLS measures the hydrodynamic diameter of the particles, which includes the hydration layer, polymeric shells, or other possible stabilizers, leading to a larger overall NP size [31]. This is because hydrodynamic size measurements obtained via DLS are obtained not only on the metal core but also with all biomolecules present in the liquid sample.

The difference in the size of both SeNPs due to the instruments used is related to the sample preparation technique and the detection system of the instruments. This difference in the average size of the SeNPs evaluated is around one order of magnitude, which could be related to their antimicrobial activity, as has been reported in other SeNPs synthesized with sodium thiosulfate and PVA, where the 81 nm SeNPs had the highest inhibition of *Staphylococcus aureus* growth compared to 124, 161, and 205 nm SeNPs, indicating that smaller SeNPs have the greatest antibacterial effect [32].

In previously performed analyses, it was described that the absorption bands around 3200 cm^−1^ for SeNPs correspond to the stretching of the -OH groups of phenolic compounds and alcohols present in the leaf extract of *A. glaucum* [23] and flower extracts of *C. officinalis* [24]. The presence of these groups could indicate their reducing role in the synthesis of SeNPs due to the interaction of Se with the -OH group via hydrogen (H) bonds [16]. One of the main differences between the SeNPs obtained is the presence of an absorption band at 2339 cm^−1^ for the SeNPs-COF that is related to the presence of C=C conjugated and C≡C [33], in addition to a band at 1741 cm^−1^ that corresponds to the C=O ester fatty acid group [33]. SeNPs-AGL is distinguished by the presence of the band at 1504 cm^−1^ (Figure 2), very close to the length of 1510 cm^−1^ reported by Mecozzi and Sturchio [33] for the lignin skeletal band (aromatic).

In addition, bands close to each other can be observed between both SeNPs, such as 1567 vs. 1621 cm^−1^, which could be attributed to the bending vibration of the carboxyl group (C=O) and 1377 vs. 1403 cm^−1^, which could be related to aromatic rings and alkanes (C-C and C-H stretching) [16], as well as the CH and CH_2_ aliphatic bending group [33]. The band corresponding to 1058 (SeNPs-AGL) and 1043 (SeNPs-COF) cm^−1^ was related to a characteristic vibration of Se-O stretching involved in the reduction of SeNPs by a reducing agent, which in the case of SeNPs-COF can be attributed to the presence of AsAc [24].

There are other bands (926, 871, 784, and 557 cm^−1^) corresponding to bending stretches that can be attributed to carboxylic groups, alkanes, and amines; the importance of these functional groups lies in their participation as different reducing and stabilizing agents during the synthesis of SeNPs [16,23,24].

The spectra resulting from the EDS analysis confirm the presence of Se, C, and O (Figure 3), indicating the formation of SeNPs. The variation in the elemental composition of the SeNPs-AGL and COF, concerning the presence of C and O, could be due to the organic compounds present in the extracts of *A. glaucum* and *C. officinalis*, mainly secondary metabolites in leaves and flowers, respectively. It has been reported that, during green synthesis, plant phytochemicals coat the surface of SeNPs, as in the case of SeNPs obtained with green orange peel extract, particularly polyphenolic compounds acting as reducing agents [17]. A raised percentage of C (Table 3) could be due to the adhesive carbon tape used to fix the sample. One of the main differences between both SeNPs is the presence of C; SeNPs-COF have almost double C mass percentage, which can be attributed to the presence of functional groups rich in C, as can be seen in the FTIR analysis (Figure 2) where SeNPs-COF have specific bands corresponding to the presence of C=C conjugated, C≡C, and C=O ester fatty acid groups [33].

There are reports on the synthesis of SeNPs. However, their use is mostly focused on combating clinical-type pathogens such as *P. aeruginosa* [34], *E. coli* [35], and *C. albicans* [36]. Therefore, the importance of the results presented here is also linked to the potential application of SeNPs to reduce crop damage caused by species of phytopathogenic fungi such as *Fusarium,* which bring economic losses and decreased plant production [37].

Regarding the antifungal activity of the NPs, it was found that both SeNPs-AGL and SeNPs-COF inhibited the growth of *F. oxysporum* (Figure 4) and *C. gloeosporioides* (Figure 5). Similar results have been described by Joshi et al. [38], in which they revealed that SeNPs synthesized biologically using *Trichoderma atroviride* inhibited the mycelial growth of a fungus of the genus *Colletotrichum*.

In the synthesis of SeNPs-COFs, ascorbic acid was used as a reducing agent (Table 2); this compound did not exhibit antifungal activity (Figure 6 and Figure 7). Similarly, Georgiou and Petropoulou have already tested the effect of ascorbic acid on fungal growth in the fungus *Rhizoctonia solani* [39], and it was likewise determined that it did not affect the growth rate of that fungus. This could be because some fungi naturally produce ascorbic acid; however, its role in the development of fungi is still unknown.

Some studies demonstrate the effectiveness of using NPs of various elements against *F. oxysporum*, such as AgNPs. This type of NP has been shown to be capable of reducing the formation of *F. oxysporum* colonies in a PDA medium in a period of 5 h under concentrations of 1 to 5 mg/mL [40]. While in this work, concentrations from 0.25 to 1.7 mg/mL were evaluated, antifungal activity was observed in all of them.

In other studies, it was reported that SeNPs had antifungal properties at lower concentrations, such as the one by El-Saadony et al. [37], in whose research different concentrations of SeNPs (50–150 µg/mL) were used, obtained via green (BioSeNPs) and chemical (CSeNPs) methods against *F. graminearum*, *F. cereales*, *F. poae*, *F. ave-naceum*, and *F. culmorum*. From this evaluation, it was found that the BioSeNPs were more effective than the CSeNPs against the tested fungi. Similarly, a lower minimum inhibitory concentration (MIC) was reported for BioSeNPs.

In turn, Joshi et al. [38] reported the antifungal activity of SeNPs under in vivo conditions using chili and tomato leaves inoculated with *C. capsici* and *Alternaria solani*. This revealed that the SeNPs inhibited mycelial growth at concentrations of 50 and 100 µg/mL, a lower concentration than that used in this research. Meanwhile, Selem et al. [41] suggest that SeNPs (synthesized with pomegranate peel extract) and chitosan nanoparticles are nanocomposite agents with effective fungicidal effects to control *P. digitatum* strains, using concentrations of 10–100 mg/mL, that is, requiring a higher concentration compared to the effects found in this research with SeNPs obtained with *A. glaucum* extracts (Figure 8 and Figure 10). This being said, the differences in the form of administration of the NPs must be taken into account. Therefore, it is ideal to take these studies to similar systems in vivo to make more appropriate comparisons.

Other studies have demonstrated the antifungal activity of SeNPs against various fungal species. In the case of Gunti et al. [42], they report that SeNPs, produced from *Emblica officinals* fruit extracts, demonstrated a wide range of antimicrobial activity against foodborne fungal pathogens (*Aspergillus brasiliensis*, *A. flavus*, *A. oryzae*, *A. ochraceus*, *F. anthophilum*, and *Rhizopus stolonifer*). In another investigation, the antifungal activity of SeNPs was tested against *Pyricularia grisea*, a fungus that causes blast disease in pearl millet, in a seven-day incubation period. On the fifth day of incubation, it was observed that very low concentrations of SeNPs (100 and 200 ppm) were able to effectively inhibit the growth of the fungus [38].

In this work, at a concentration of 0.25 mg/mL, a differential effect was found among the SeNPs evaluated. SeNPs-AGL had a higher inhibitory effect against *F. oxysporum* than SeNPs-COF after day 10 (Figure 8 and Figure 9). A similar result was obtained against *C. gloeosporioides*; this pathogenic fungus demonstrated greater sensitivity to SeNPs-AGL (Figure 10 and Figure 11). This behavior can be attributed to the phytochemical composition of the extracts used to synthesize SeNPs, as well as a smaller size of the SeNPS-AGLs (Figure 1), since, as mentioned above, smaller NPs have higher antimicrobial activity [32].

This is explained by the fact that smaller-size nanoparticles have higher stability and a high surface area/volume ratio, which results in their antimicrobial properties that are not observed in the same material on a larger scale in its bulk form [18]. These two characteristics of NPs (smaller size and high surface area/volume ratio) allow them to interact closely with the cell membranes of microorganisms, which facilitates interactions and intracellular diffusion, triggering considerable damage to membranes and causing toxic effects on DNA [43] or inhibiting cell proliferation by reactive oxygen species (ROS)-mediated processes [18]. In addition, it has been proposed that the antimicrobial activity of NPs may be due to the release of ions into the medium where microorganisms grow. It has been indicated that ion dissolution depends on the size of the NPs and the concentration of the solution [44]. In the case of SeNPs, research indicates that these NPs have size- and concentration-dependent antimicrobial effects against various microorganisms [45].

In *F. oxysporum*, an alteration induced by SeNPs in the morphology of the fungal colonies was observed. In particular, a slightly cottony texture and a lower mycelium density were observed compared to the control (Figure 8 and Figure 9). Likewise, using SeNPs produced a reddish pigmentation in the area lateral to the mycelial disc of *F. oxysporum*.

Morphological changes also occurred in the *C. gloeosporioides* colonies (Figure 10 and Figure 11). Although mycelial growth was observed, it was found that this was different from that presented by the control. For example, the amount of mycelium and the cottony shape of the fungus were reduced. Similarly, rough-looking tissue was formed at the base of *C. gloeosporioides*, in contrast to the smooth growth achieved with the fungus without SeNPs.

SeNPs have previously shown high antifungal activity through the inhibition of spore germination [38]. Pigmentation changes could indicate the presence of compounds responsible for reducing the antifungal effect achieved by SeNPs. Changes in the morphology and coloration of the genus *Fusarium* have been reported by Troni et al. [46] with the use of different forms of Se (Na_2_SeO_3_, Na_2_SeO_4_, SeMet, and SeCys) on *F. proliferatum*. At the same time, during the evaluation of the mycelial growth, a strong odor was observed in the culture medium containing the SeNPs. This change could be due to the production of volatile compounds, such as dimethyl selenide (CH_3_)_2_Se, as a fungal mechanism invoked to reduce the toxicity caused by SeNPs [47]. The latter could also indicate the presence of mycotoxins typical of fungi of the *Fusarium* genus, such as zearalenone, trichothecenes, deoxynivalenol, and fumonisins, all of which are produced by the fungus as a secondary defense mechanism to protect itself from excess ROS elicited by SeNPs [48].

On the other hand, there is a growing concern about the health and environmental problems generated by chemical residues applied during crop production due to the excessive use of synthetic/chemical fungicides because of acute toxicity, prolonged degradation periods, and the emerging fungal resistance of phytopathogenic microorganisms [14,41]. SeNPs obtained via green synthesis are gaining attention as a safe, effective, and environmentally friendly alternative, as they represent a lower risk to personnel applying such products and to the environment. Both SeNPs-AGL and SeNPs-COF can be used to manage postharvest fungal infections, particularly for the control of *C. gloeosporioides*, a fungus that affects the postharvest quality of avocado, a fruit of high economic importance. These SeNPs are also efficient in inhibiting the growth of *F. oxysporum*, a cosmopolitan fungus that causes lethal diseases in a wide range of highly profitable crops. Foliar application of these SeNPs could be an option as defense inducers against fungal diseases or as elicitors of the plant defense system; their application to the root could also be considered in the integrated management of plant diseases. Finally, the application of low concentrations (<50 µM) of these SeNPs could result in biostimulation of plant growth, as was obtained with SeNPs-AGL [23,31].

## 4. Materials and Methods

### 4.1. Plant Material

In the case of calendula, flowers of the Costa Orange variety grown in a greenhouse were used. In the case of *A. glaucum*, leaves collected in La Huerta, Jalisco (19°29′24.2″ N 105°02′33.9″ W were used. Plant material was preserved until use in an ultra-freezer (Forma™ 900 Series, ThermoFisher, Waltham, MA, USA) at a temperature of −80 °C.

### 4.2. Preparation of the Extract

For five days, the frozen samples were lyophilized in a freezer dryer (Freezone equipment, Labconco, Kansas, USA). The dried plant tissue samples were pulverized in an industrial mill (MF10.1BS1, IKA, Staufen, Germany) until a fine particle size was obtained. All extractions were carried out using methanol with variations in the proportion and homogenization method. *A. glaucum* leaf extract was obtained via maceration with methanol in a 1:10 (*w/v*) ratio for 24 h. For the calendula flowers, the extraction was carried out using methanol (1:20 *w/v*) and homogenization assisted with ultrasound, using an ultrasonic bath of 100 W maximum nominal output power and frequency of 42 kHz (2510R-DTH, Branson Ultrasonics, Danbury, CT, USA), for three times of 15 min at a constant temperature. (25 °C).

At the end of each extraction, the liquid extract was obtained by eliminating the insoluble plant biomass via filtration with Whatman filter paper with a pore size of 6–11 µm. Finally, the solvent was evaporated under reduced pressure using a rotary evaporator (R-100, Buchi, Flawil, Switzerland) at a bath temperature of 40 °C.

The extracts obtained were stored in amber vials at −80 °C and subsequently lyophilized to be obtained.

### 4.3. Evaluation of the Antimicrobial Activity of Plant Extracts

The antifungal activity of methanolic extracts of flowers (COF) of *C. officinalis*, as well as methanolic extracts of leaves of *A. glaucum*, were evaluated using the Kirby–Bauer method. A suspension of spores/hyphal fragments of the strains of the phytopathogenic fungi was prepared (Table 4), for which the propagules were collected and poured into a glass test tube with 6 mL of sterile distilled water and glass beads to homogenize the solution in a vortex shaker. The inoculum solution was homogenized and filtered using a sterile sieve (106 μm). The homogeneous spore mixture was quantified using a Neubauer chamber.

For the evaluation of the activity of plant extracts, 100 μL of the propagule of the phytopathogenic fungi was deposited on PDA agar plates. Subsequently, the suspension was homogeneously distributed in the culture medium with a Drigalski loop. Whatman #1 filter paper discs of 6 mm diameter were prepared, to which 20 μL of the extract to be evaluated was previously added (10 μL on each side of the disc). Methanol was included as a negative control and Benomyl (BEN) as a positive control. Plant extract concentrations of 50 and 500 mg/mL were evaluated. The discs were placed on a completely dry medium. The Petri dishes were incubated at 37 °C until the growth of the tested fungal species was observed. The inhibition diameter of each disc was determined manually and digitally using ImageJ software.

For the antibacterial activity, the methodology described by Hernández-Díaz et al. [24] was followed, using the bacterial strains *Serratia marcescens*, *Enterobacter cloacae*, and *Alcaligenes faecalis*.

### 4.4. SeNPs Synthesis

The synthesis of SeNPs-AGL was performed as reported by Garza-García et al. [23]. Extracts obtained from *A. glaucum* leaves (50 mg/mL) were used, adding a total volume of 80 μL in 10 mL of 10 mM Na_2_SeO_3_ (Sigma-Aldrich, St. Louis, MO, USA). The reaction was carried out with magnetic stirring (1200 rpm) at 40 °C for 40 min. The reaction was completed in the dark with magnetic stirring and room temperature for 24 h. For the synthesis of SeNPs-COF, we followed the indications of Hernández-Díaz et al. [24], using extracts of *C. officinalis* flowers (100 mg/mL). We used 10 mL of Na_2_SeO_3_ solution (10 mM), a final volume of 120 μL of COF extract, and a total of 3.5 mL of ascorbic acid (AsAc) (40 mM). The reaction was carried out at 40 °C under stirring (1150 rpm) for 35 min. The reaction was completed in the dark at 4 °C for 24 h.

### 4.5. Determination of the Size of the Nanoparticles

The SeNPs were sonicated for 1 min, diluted in water (1/1000), and Tween-20 surfactant (20 µL) was added. Subsequently, the samples were analyzed in a particle analyzer (Litesizer 500, Anton Paar, Graz, Austria).

### 4.6. Fourier Transform Infrared Spectroscopy (FTIR) Analysis of SeNPs

FTIR analysis of SeNPs-AGL and COF was carried out on the Spectrum Two FTIR Spectrometer (UATR Two, PerkinElmer, Beaconsfield, UK) in the 4000–500 cm^−1^ region. OriginPro 2022 software (OriginLab Corporation, Northampton, MA, USA) was used to prepare the plots.

### 4.7. Determination of the Elemental Composition of NPs

The SeNPs were frozen at −80 °C and lyophilized to dryness (Freezone equipment, Labconco, Kansas City, MO, USA). The lyophilized sample was pulverized in a ceramic mortar and then placed on copper tape. Samples were analyzed under a scanning electron microscope (JEOL JSM-6010LA, Tokyo, Japan) using an integrated energy dispersive X-ray spectroscopy analyzer (EDS).

### 4.8. Fungal Strains

**Figure 12:**
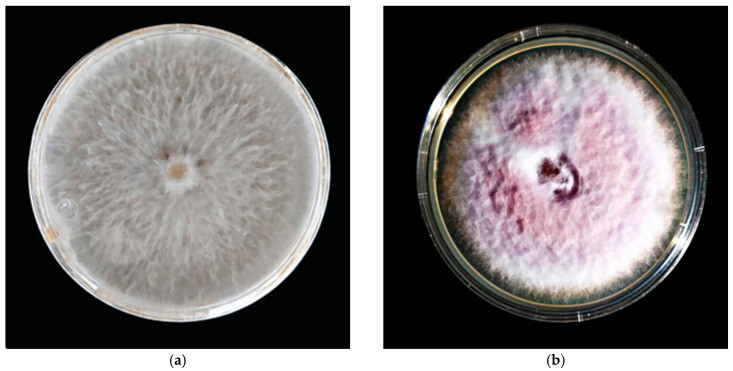
Characteristic mycelial growth at 15 d in PDA medium. (**a**) *C. gloeosporioides* and (**b**) *F. oxysporum*.

Two fungal strains isolated from the avocado fruit (*Persea americana*) were used. Identification of fungal strains was performed by an alignment in the Basic Local Alignment Search Tool (Available at https://www.ncbi.nlm.nih.gov/, accessed on 08 September 2021), using as input the consensus sequence of the amplicon corresponding to the internal transcribed space (ITS) of the ribosomal DNA. The strains were identified as *F. oxysporum* (HQ647333.1, 99.45% identity) and *C. gloeosporioides* (MT254840.1, 99.82% identity) (Figure 12). The consensus nucleotide sequence was deposited in GenBank under accession numbers OK067244.1 (*F. oxysporum*) and OK067243.1 (*C. gloeosporioides*).

### 4.9. Evaluation of the Antifungal Activity of SeNPs

The antifungal evaluation of the SeNPs was carried out using the inhibition method for the mycelial growth of *F. oxysporum* and *C. gloeosporioides* in a medium poisoned superficially with SeNPs and their components separately, as well as by SeNPs embedded in the medium. For the first case, PDA Petri dishes were prepared, to which the different treatments were added, such as 1 mL of SeNPs (1.7 mg/mL for *A. glaucum* and 1.28 mg/mL for *C. officinalis* of 10 mM Na_2_SeO_3_ initial solution), 1 mL of the commercial broad-spectrum antifungal cycloheximide (CHX) (2.5 mg/mL), and the respective controls, including ascorbic acid, in the test, spreading them in the Petri dish plate. Finally, a mycelial disk of axenic fungal cultures (7 mm) with a growth of 6 days was deposited. The inoculated dishes were incubated in a natural convection incubator (Precision Scientific, 818, Chennai, India) at room temperature for 15 days. Colony diameter was measured at 4, 7, 10, and 15 days after setting up the experiment.

For a second assay, different concentrations of SeNPs (0, 0.25, 0.5, and 1 mg/mL) were embedded in the PDA medium without first solidifying. The medium was poured into Petri dishes and allowed to solidify. In the same way, mycelial discs of axenic fungal cultures (7 mm) with a growth of 6 days were placed in the center of the PDA Petri dishes, and the same incubation and measurement methodology described for the previous procedure was followed. All treatments were performed in triplicate.

### 4.10. Statistic Analysis

For antifungal activity, a two-way analysis of variance (ANOVA) was performed, and the separation of means was obtained with the procedures of SAS 9.1 statistical software (SAS Institute, Cary, NC, USA), using Duncan’s multiple range test with a significance level of *p* < 0.05.

## 5. Conclusions

Methanolic extracts of *A. glaucum* leaves (AGL) and *C. officinalis* flowers (COF) had antimicrobial activity. The AGL extracts demonstrated antifungal activity against *C. cladosporioides* and antibacterial activity against *S. marcescens*, *E. cloacae*, and *A. faecalis*. Meanwhile, COF extracts only had antibacterial activity against *S. marcescens*.

SeNPs obtained via green synthesis using AGL and COF methanolic extracts had different characteristics in terms of size, presence of functional groups, and elemental composition. SeNPs-AGl had a size of about 8.0 nm and SeNPs-COF a size of 134 nm. FTIR analysis confirmed the presence of different functional groups in both SeNPs, with which was found a variable elemental composition mainly of C and Se.

Both SeNPs demonstrated antifungal activity against two plant pathogenic fungi (*F. oxysporum* and *C. gloeosporioides*) that limit commercial crop production. The lowest evaluated concentration of SeNPs (0.25 mg/mL) allowed confirming the correlation between the size of the NPs and their antimicrobial activity, where the smallest SeNPs-AGLs presented the highest antifungal activity for both *F. oxysporum* and *C. gloeosporioides*. This antifungal effect of SeNPs may also be attributed to the phytochemical composition of the extracts used for green synthesis, as AGL extracts demonstrated higher antimicrobial activity than COF extracts.

For future trials, it is necessary to include lower concentrations of SeNPs in both in vitro and in vivo cytotoxicity assays to determine the possible deleterious effects of these SeNPs, in addition to exploring and understanding the possible mechanisms of action of the antimicrobial activity of SeNPs. Because of their antifungal activity, these SeNPs could potentially be used in the agricultural, pharmaceutical, or biomedical industries.

## 6. Patents

This work resulted in two patent applications presented to the “Instituto Mexicano de la Propiedad Industrial” (Mexican Institute of Industrial Property): application MXa2021015367 for SeNPs-AGL and application MXa2022011926 for SeNPs-COF.

## Figures and Tables

**Table 1:** Inhibition halos (mm) generated by the antimicrobial effect of *A. glaucum* leaf (AGL) and *C. officinalis* flower (COF) extracts.

Pathogenic Microorganism	Plant Extracts		Positive Control	
	AGL	COF ^1^	BEN	GEN/CIP ^2^
*F. oxysporum*	0.00	0.00	6.49 + 0.34	-
*C. gloeosporioides*	0.00	0.00	5.29 + 0.17	-
*C. cladosporioides*	9.32 ± 0.23	0.00	7.18 ± 0.18	-
*S. marcescens*	9.24 ± 0.23	6.14 ± 0.15	-	9.23 ± 0.23
*A. faecalis*	9.74 ± 0.24	0.00	-	10.08 ± 0.25
*E. cloacae*	9.49 ± 0.23	0.00	-	9.80 ± 0.24

AGL: *A. glaucum* extracts at a concentration of 500 mg/mL for fungi and 300 mg/mL for bacteria. COF: *C. officinalis* flower extracts at a concentration of 500 mg/mL for fungi and 300 mg/mL for bacteria. BEN: benomyl. GEN: gentamycin. CIP: ciprofloxacin. ^1^: The antibacterial activity data were taken from Hernández-Díaz et al. [24]. ^2^: CIP was used as a positive control in the *A. faecalis* assay.

**Table 2:** Size of selenium nanoparticles (SeNPs) synthesized with extracts of different plant species.

Nanoparticles	Plant Source	Plant Tissue	Reducing Agent	Stabilizing Agent	Size (nm)
SeNPs-AGL	*A. glaucum*	Leaves	Leaves extracts	Leaves extracts	8.08
SeNPs-COF	*C. officinalis*	Flowers	Ascorbic acid	Flower extracts	133.98

**Table 3:** Elemental composition via Litesizer of the selenium nanoparticles (SeNPs) synthesized with extracts of *A. glaucum* (SeNPs-AGL) and *C. officinalis* (SeNPs-COF).

Chemical Formula	SeNPs-AGL		SeNPs-COF	
	Mass (%)	Mol (%)	Mass (%)	Mol (%)
Se	31.27	8.13	19.11	4.03
C	24.39	41.71	46.68	64.71
O	27.49	35.29	21.43	22.30
Na	16.05	14.34	11.73	8.49
Si	0.52	0.38	-	-
Cl	-	-	0.49	0.23
K	0.29	0.15	0.56	0.24
Total	100.00	100.00	100.00	100.00

**Table 4:** Bioassay conditions with the plant pathogenic fungi evaluated.

Pathogenic Fungi	Propagule	Concentration	Benomyl Concentration (mg/mL)
*F. oxysporum*	Conidia	1 × 10^5^	35
*C. gloeosporioides*	Conidia	1 × 10^4^	35
*C. cladosporioides*	Conidia	5 × 10^4^	20

## Data Availability

Not applicable.
